# Determination risk factors for severe and profound hearing loss in child candidates for cochlear implantation in southeast of Iran during 2014-2020

**DOI:** 10.1186/s12887-022-03124-5

**Published:** 2022-01-26

**Authors:** Parya Jangipour Afshar, Jila Afsharmanesh, Marzieh Eslahi, Hojjat Sheikhbardsiri, Mahmood Nekoei Moghadam

**Affiliations:** 1 Department of Biostatistics and Epidemiology, Faculty of Public Health, Kerman University of Medical Sciences, Kerman, Iran; 2 Neurology Research Center, Kerman University of Medical Science, Kerman, Iran; 3 Department of Biostatistics and Epidemiology, School of Public Health, Kerman University of Medical Sciences, Kerman, Iran; 4 Health in Disasters and Emergencies Research Center, Institute for Futures Studies in Health, Kerman University of Medical Sciences, Kerman, Iran

**Keywords:** Risk factors, Severe and profound deafness, Cochlear implantation

## Abstract

**Background:**

Hearing loss can have a major impact on children’s language development, academic success and hearing comprehension. The aim of the present study was to determinate risk factors for severe and profound hearing loss in child candidates for cochlear implantation in southeast of Iran during 2014–2020.

**Materials and methods:**

This case-control study consisted of 400 children referring to a cochlear implant center (in southeastern Iran) from Bandar Abbas, Zahedan and Kerman during the years 2014–2020 as cases. The subjects were selected using the random sampling method; 200 children hospitalized in Shafa and Afzalipour hospitals were selected as controls.

**Results:**

Based on the results of the multivariate logistic regression, weight less than 1500 g (OR = 4.40: *p* < 0.05), hospitalization in NICU (OR = 7.21: *p* < 0.05), family history of hearing loss (OR = 11.47: *p* < 0.05), Gestational age over 35 (OR = 9.63: *p* < 0.05), intracranial hemorrhage (OR = 5.18: *p* < 0.05), consanguineous marriage (OR = 12.48: *p* < 0.05) and high fever and seizures (OR = 3.02: *p* < 0.05) were recognized as risk factors for sensorineural deafness in children.

**Conclusion:**

Most of the risk factors for deafness are preventable, and hereditary factors play an important role in congenital deafness in children. Therefore, genetic counseling before consanguineous marriage, early diagnosis, timely intervention can prevent many cases of hearing loss in children.

## Introduction

Hearing is known to be the main sensory access of human at the beginning of life which enables him to identify, pay attention, trace sounds and integrate essential hearing experiences to develop speech and language [1]. The integrity of the auditory system in children is crucial to appropriate development of oral language, perceive their surroundings, interact with peers, organize and develop thoughts and feelings, and gain knowledge [2]. Hearing impairment is defined as a decreased ability to notice sound with structural or functional deviation from normality [2]. The American Speech-Language-Hearing Association (ASHA) defined several levels for hearing loss (HL): the Mild HL, ranges between 26 to 40 dB; the moderate HL, ranges between 41 to 55 dB; the moderate to severe HL, ranges between 56 to 70 dB; severe or profound HL, ranges between 71 to 90 dB and the profound HL, range is above 91 dB [3]. People with severe to profound hearing loss are considered deaf, and those with mild to moderate hearing loss are considered hard of hearing [4]. Deafness is the most common neurological disorder worldwide, affecting more than half a billion people [4, 5]. Hearing deficiency represents a public health problem due to its impact on the citizen [2]. The prevalence of sensorineural hearing loss (SHL) in developed countries is estimated to be 1–3 of every 1000 children born. This rate is supposed to be higher in developing countries [6]. Infants’ hearing loss may occur before, during or after birth due to several reasons [1]. Family history of hearing deficiency, intrauterine infections, neonatal disorders, persistence pulmonary hypertension (PPHN) associated with mechanical ventilation and after birth infection like bacterial meningitis, are well established risk factors of hearing loss in children [7]. Evidences indicate that approximately 22 to 35% of children with hearing loss fail at least one grade and up to 33% of children with hearing loss will progress this disorder. In addition, behavioral attention-deficit hyperactivity disorder-type problems are identified in 20% of children with hearing loss [8]. Delayed diagnosis of hearing loss in children and delayed access to early intervention programs may worsen the negative consequences for development of language, cognitive, and social-emotional skills [9]. Hearing loss related complications in children necessitates the need for early detection and intervention strategies [1]. Regarding the advances in care and new scientific knowledge during recent years, it is necessary to update risk factors to reflect current clinical practice [9]. There are limited evidences on the magnitude of hearing loss in Iran and most of them are not up-to-date [10]. To the best of our knowledge, there are limited evidences on the risk factors of hearing loss in Iran. Previous studies in this field had limited sample size and limited study period [10, 11]. So the present study is the first comprehensive study on hearing loss risk factors in children southeast of Iran.

## Materials and methods

This case-control study aimed to elucidate the role of risk factors related to hearing loss and deafness in children. The case sample consisted of 400 children referring to a cochlear implant center (in southeastern Iran) from Bandar Abbas, Zahedan and Kerman during the years 2014–2020. The subjects were selected using the random sampling method; 200 children hospitalized in Shafa and Afzalipour hospitals were selected as hospital controls.

Inclusion criteria for the case group were diagnosis of severe (not hearing sounds between 71 and 90 dB) and profound hearing loss (not hearing sounds more than 91 dB) by the physician or undergoing cochlear implantation.

The controls of this study consisted of children hospitalized in other wards or outpatient clinics of Shafa and Afzalipour hospitals who had no hearing loss or deafness at the time of the study and had no history of this disease in their past. Data was collected through face-to-face, telephone interviews and medical records using a questionnaire. This questionnaire contained information about age, gender, consanguineous marriage and family history that collected from interviews. The other information about medical care gathered from medical records. In order to comply with the principles of medical ethics, oral consent was obtained from patients that interviewed by telephone and written consent was obtained from patients interviewed through face-to-face. Univariate and multivariate logistic regression models were used to analyze the data, and the risk of hearing loss or deafness was expressed as odds ratio calculations and estimates (95% confidence). First, univariate regression was used; the significant variables were then entered into multivariate regression. Data was analyzed using SPSS software (version 24). *P*-value of less than 0.05 was considered significant.

## Results

In this study, 600 children were examined (400 hearing impaired and deaf children in the case group and 200 healthy ones in the control group). Out of 400 children, 216 were boys (54.5%) and 184 were girls (46.0%), with the average age of 8.91 ± 4.39. In the control group, 93 were boys (46.5%) and 107 were girls (53.5%), with the average age of 6.73 ± 3.26.

Univariate logistic regression was first used to examine the effective factors; all variables (except for gestational diabetes (*p* > 0.05)) were significant factors affecting sensory-neurological deafness in children (Table 1). In the next step, since factors identified as the risk of deafness certainly affected each other, the simultaneous multivariate logistic regression method was used to control the impact of other factors, and the effect of each factor was examined independently by assuming the constancy of other factors. Therefore, all variables were entered into the logistic regression. According to the multivariate logistic regression model, weight less than 1500 g (OR = 4.40: *p* < 0.05), hospitalization in NICU (OR = 7.21: *p* < 0.05), family history of hearing loss (OR = 11.47: *p* < 0.05), Gestational age over 35 (OR = 9.63: *p* < 0.05), intracranial hemorrhage (OR = 5.18: *p* < 0.05), consanguineous marriage (OR = 12.48: *p* < 0.05) and high fever and seizures (OR = 3.02: *p* < 0.05) were recognized as risk factors for sensorineural deafness in children (Table 2).

**Table 1:** Characteristic of the study population and odds ratios using logistic regression for all variables

variable	Case ***n*** = 400	Control ***n*** = 200	Odds ratio (95% CI)	***P***-value
**weight less than 1500 g**				
No	344	191	1	0.026
Yes	38	9	2.34(1.11,4.95)	
**hospitalization in NICU**				
No	340	194	1	< 0.001
Yes	59	6	5.61(2.37,13.23)	
**hypoxia**				
No	341	179	1	0.039
Yes	59	17	1.82(1.03,3.21)	
**Bilirubin more than 10**				
No	306	184	1	< 0.001
Yes	94	16	3.53(2.01,6.18)	
**Gestational age over 35**				
No	348	197	1	< 0.001
Yes	46	3	8.68(2.66,28.27)	
**Gestational diabetes**				
No	383	196	1	0.167
Yes	17	4	2.17(0.77,6.552)	
**Meningitis**				
No	382	198	1	0.040
Yes	18	2	4.66(1.07,20.30)	
**high fever and seizures**				
No	304	182	1	< 0.001
Yes	96	18	3.19(1.86,5.45)	
**family history of hearing loss**				
No	235	195	1	< 0.001
Yes	165	5	27.38(11.02,68.01)	
**consanguineous marriage**				
No	124	174	1	< 0.001
Yes	276	26	14.89(9.37,23.67)	
**congenital infection (TORCH)**				
No	379	198	1	0.022
Yes	21	2	5.48(1.27,23.63)	
**intracranial hemorrhage**				
No	224	187	1	< 0.001
Yes	176	13	11.30(6.22,20.50)	

The sum of subgroups may be less than total because of missing data

**Table 2:** Multivariate logistic regression adjusted regression for variables in the table

variable	Odds ratio	***P***-value	(95% CI)
weight less than 1500 g	4.40	0.025	(1.20,16.13)
hospitalization in NICU	7.21	< 0.001	(6.11,44.40)
family history of hearing loss	16.47	< 0.001	(6.11,44.40)
Gestational age over 35	9.63	< 0.001	(2.47,37.48)
intracranial hemorrhage	5.18	< 0.001	(2.52,10.65)
consanguineous marriage	12.48	< 0.001	(7.29,21.37)
high fever and seizures	3.02	< 0.001	(1.50,6.07)
constant	0.20		

There was no significant 2 by 2 interaction between variables (not shown in the table)

## Discussion

Our case-control study revealed several risk factors of developing hearing loss in southeast of Iran, birth weight lower than 1500 g, being hospitalized in NICU, family history of hearing loss, pregnancy at the age of 35 or higher ages, intracranial hemorrhage, consanguinity, high fever and seizures. Studies with populations of children with low birth weight have shown rates of prevalence and relative risk of developing hearing loss which were inversely proportional to the weight quantified at birth [12]. Our study confirmed the role of birth weight as a risk factor for hearing loss. Similar to findings of our study, Pruszewicz et al. indicated that the greatest risk of the acquired profound hearing loss and deafness occurring in low birth weight children is connected with the general physical status of the neonates and the treatment program in the neonatal intensive care unit [13]. In this regard, Engdahla et al. also showed that the risk of sensorineural hearing loss increased with decreasing birth weights. Low birth weight increased the risk of both moderate and severe/profound sensorineural hearing loss, suggesting no differential impact of birth weight on the severity of hearing loss. The study also revealed that length of gestation had no independent impact, suggesting intrauterine growth restriction as a major mechanism of sensorineural hearing loss [14]. Dimopoulou et al. showed that in asymptomatic congenitally CMV-infected infants, birth weight and low birth weight are associated with sensorineural hearing loss [15].

It has been observed that hearing loss is more prevalent in newborns admitted to the Neonatal Intensive Care Unit (NICU) [16]. A systematic review and meta-analysis by Butcher et al. showed that prevalence of permanent childhood hearing loss was 6.9 times higher among those admitted to NICU [17]. Similar to a study by Reis et al. [1] we observed that being hospitalized in NICU is associated with higher risk of hearing loss. In a cross-sectional study on 530 neonates admitted to NICU Abuzar Hospital in Ahvaz, Iran, 5.09% were diagnosed with different types of hearing loss. Findings of the study revealed that hearing loss is associated with antibiotic therapy [16]. Recchia et al. also suggested that use of ototoxic drugs is one of the causes of hearing loss in infants hospitalized in the NICU [18]. In a study by Keihanidost et al. on neonates older than 6 months with history of hospitalization in intensive care unit, neonatal icterus associated with phototherapy, respiratory distress syndrome (RDS) and lower Apgar score were noted as risk factors for hearing loss [7]. In addition, a nationwide cohort in Netherlands on prevalence and independent risk factors for hearing loss in NICU infants showed that severe birth asphyxia and assisted ventilation ≥5 days were risk factors of hearing loss [19]. It can be concluded that infants who are hospitalized in NICU are more vulnerable to hearing loss mostly because of underlying causes such as adverse effects of premature birth and related medications.

The Joint Committee on Infant Hearing has noticed family history of hearing loss as risk factor for permanent congenital, delayed onset, or progressive hearing loss since 1973 [20]. In our study we confirmed the role of family history of hearing loss as a risk factor. Similarly, Kataoka et al. stated that hearing loss was the most frequent risk factor and was observed for 30.2% of all patients with delayed-onset hearing loss in early childhood [21]. Gouveia et al. described unilateral and bilateral asymmetric sensorineural hearing loss in a group of Spanish children and suggested that family history of permanent deafness, which began in childhood is the highest risk indicator for hearing loss [22]. Another study in Spain investigated the prevalence of hearing loss and results of newborn hearing screening and audiological diagnosis in private health care systems and suggested that of the 160 patients identified as having high risk for hearing loss, 17.2% had a family history of hearing loss in childhood [23]. A retrospective study from 2010 to 2014 on children with confirmed hearing loss identified through universal newborn hearing screening (UNHS) in Virginia revealed that family history was the most common risk factors in bilateral hearing loss [8]. It seems that family history of hearing loss is a strong indicator of hearing loss in children.

Consanguinity is common in some regions of Asia and Africa because of socioeconomic, cultural, and religious factors. Historical studies report a strong preference consanguineous marriages in Iranians. Consanguineous marriage is an important risk factor of genetic disorders [6]. Our study confirmed the role of consanguinity as a risk factor for hearing loss in children. Ajallouyan et al. conducted a study to demonstrate the causes of profound bilateral sensorineural hearing loss among Iranian samples who are candidates for cochlear implantation and showed that 65% of the patients’ parents had consanguineous marriages. The study indicated that hereditary was identified as the most common cause in 33% of the patients [6]. Similarly A significant relationship was reported between hearing loss and consanguinity by Al-Gazali in United Arab Emirates [24] and Girotto et al. in Qatar [25]. Bener et al. also noted more prevalence of hearing loss in children with parental consanguinity [26]. However, Kavitha et al. study showed normal cochlear morphology in all the children born out of consanguineous marriages. Authors suggested that consanguinity, as a risk factor for development of sensorineural hearing loss, may not result in gross structural anomaly of cochlea [27].

Our study revealed that pregnancy at the age of 35 and higher is associated with increased risk of hearing loss in child. Leslie et al. also reported maternal age as a risk factor for hearing loss [28]. However Dimopoulou et al. revealed that maternal age do not affect the risk of hearing loss in asymptomatic congenitally CMV-infected newborns [15]. It seems that the role of maternal age at pregnancy in relation to children hearing loss should be studied more.

A pontine intracranial hemorrhage (ICH) evokes several neurological symptoms, due to the various nuclei and nerve fibers; however, hearing loss from a pontine ICH is rare [29], however we observed that intracranial hemorrhage is associated with increased risk of hearing loss.

Our study also revealed that fever and seizures is associated with increased risk of hearing loss in children. This finding was similar to the results of studies by Karanja et al. [30]. Murray et al. also showed that clinical seizure activity prior to neonatal extracorporeal membrane oxygenation and the duration of neonatal extracorporeal membrane oxygenation therapy are independently associated with sensorineural hearing loss [31].

## Conclusion

Hereditary factors play an important role in congenital deafness in children especially in families with a family history of hearing loss and genetic counseling prior to consanguineous marriage, which is common in Asian countries, are crucial factors to be considered. In addition, early diagnosis, timely intervention, vaccination to prevent meningitis, increased birth care and preventing the use of ototoxic medications can prevent many cases of hearing loss in children and may dramatically reduce the cost of education related to hearing loss.

## Limitation

The present study had some limitations. First, Incomplete medical records of some patients. Second Lack of parental responsibility and due to the data were collected retrospectively, which might cause to recall bias. Third, Change patients’ phone number so we couldn’t contact them.

## Data Availability

The data sets generated during the current study are available from the corresponding author.
